# Adenovirus core protein V reinforces the capsid and enhances genome release from disrupted particles

**DOI:** 10.1126/sciadv.ade9910

**Published:** 2023-04-07

**Authors:** Natalia Martín-González, Alfonso Gómez-González, Mercedes Hernando-Pérez, Michael Bauer, Urs F. Greber, Carmen San Martín, Pedro J. de Pablo

**Affiliations:** ^1^ Departament of Condensed Matter Physics, Universidad Autónoma de Madrid and Institute of Condensed Matter Physics (IFIMAC), 28049 Madrid, Spain.; ^2^ Department of Molecular Life Sciences, University of Zurich, CH-8057 Zurich, Switzerland.; ^3^ Department of Macromolecular Structures, Centro Nacional de Biotecnología (CNB-CSIC), 28049 Madrid, Spain.

## Abstract

Out of the three core proteins in human adenovirus, protein V is believed to connect the inner capsid surface to the outer genome layer. Here, we explored mechanical properties and in vitro disassembly of particles lacking protein V (Ad5-ΔV). Ad5-ΔV particles were softer and less brittle than the wild-type ones (Ad5-wt), but they were more prone to release pentons under mechanical fatigue. In Ad5-ΔV, core components did not readily diffuse out of partially disrupted capsids, and the core appeared more condensed than in Ad5-wt. These observations suggest that instead of condensing the genome, protein V antagonizes the condensing action of the other core proteins. Protein V provides mechanical reinforcement and facilitates genome release by keeping DNA connected to capsid fragments that detach during disruption. This scenario is in line with the location of protein V in the virion and its role in Ad5 cell entry.

## Teaser

Adenovirus core protein V provides mechanical stabilization and genome decondensation.

## INTRODUCTION

Some DNA viruses such as polyomaviruses use histone proteins to protect their genome and evade detection (*1*), while others, like adenoviruses (*2*, *3*), chloroviruses, and marseillevirus (*4*–*6*), encode their own histone-like proteins for condensing their genetic material within the capsid. Although adenoviruses are the most studied system encoding their own histone-like proteins, little is known about the mechanisms used to package and release their genome during assembly and uncoating.

Adenoviruses are nonenveloped viruses with a 95-nm-diameter icosahedral capsid, composed of two major coat proteins: hexon and penton base and the associated protruding protein fiber. The capsid contains additional proteins (IIIa, VIII, VI, and IX in the mastadenoviruses) that play different roles in stabilization, assembly, maturation, and entry (*2*). Inside the human adenovirus type 5 (Ad5) capsid, the 36-kbp double-stranded DNA (dsDNA) genome is bound to positively charged viral proteins that constitute ∼50% of the core molecular weight: 150 copies of protein V (41 kDa), 500 to 800 copies of protein VII (22 kDa), and 100 to 300 copies of protein μ (9 kDa) (*7*–*9*). Proteins VII and μ are cleaved by the viral protease during maturation (*10*).

The adenovirus capsid has icosahedral symmetry, and its structure has been solved at high enough resolution to trace the polypeptide chains with a high degree of confidence (*2*). In contrast, the nucleoprotein core appears disordered, hindering its structural characterization. Early electron microscopy on cores extracted from disrupted particles suggested that protein VII forms a nucleosome-like structure wrapping the viral genome, while protein V was proposed to occupy the internucleosome space (*11*). The appearance of thick fibers in cores released from immature adenovirus particles prompted the hypothesis that the precursor of protein μ could be condensing the dsDNA by a bridging mechanism (*12*, *13*). More recent studies with mechanically disrupted virions showed that protein VII appears in clusters with the DNA, adjacent to fibers that could correspond to bundles of dsDNA chains condensed by the bridging function of μ (*14*). The condensing action of protein VII is not required for genome packaging (*15*). However, the major core protein has an unexpected role in facilitating maturation of the membrane-lytic protein VI, and its presence is required for exposing protein VI allowing viral rupture of the endosomal membrane (*16*, *17*). Nevertheless, protein VII–mediated condensation appears to modulate the virion internal pressure and genome diffusion out of disrupted capsids (*14*).

Proteolytic cleavage of proteins VII and μ during Ad5 maturation along with cleavage of other virion precursor proteins and the L1 52/55 kDa packaging protein (*18*) results in an increase of internal pressure and has been proposed to facilitate penton destabilization, an important step in the stepwise virion uncoating program (*13*, *19*). Evidence for this scenario has been derived from the mechanical properties of the Ad2 *ts1* mutant (*13*, *20*, *21*). Ad2 *ts1* has a softer shell than the wild type, fails to package the viral protease due to a single point mutation in the protease (*22*), and does not cleave the precursor proteins pVII and pre-μ (*10*, *20*, *21*, *23*, *24*). Intriguingly, incoming ts1 particles do not shed the fibers and fail to penetrate through the endosomal membrane, consistent with the notion that destabilized pentons are involved in activating the membrane lytic machinery of the virion (*22*, *25*).

**Fig. 1.**
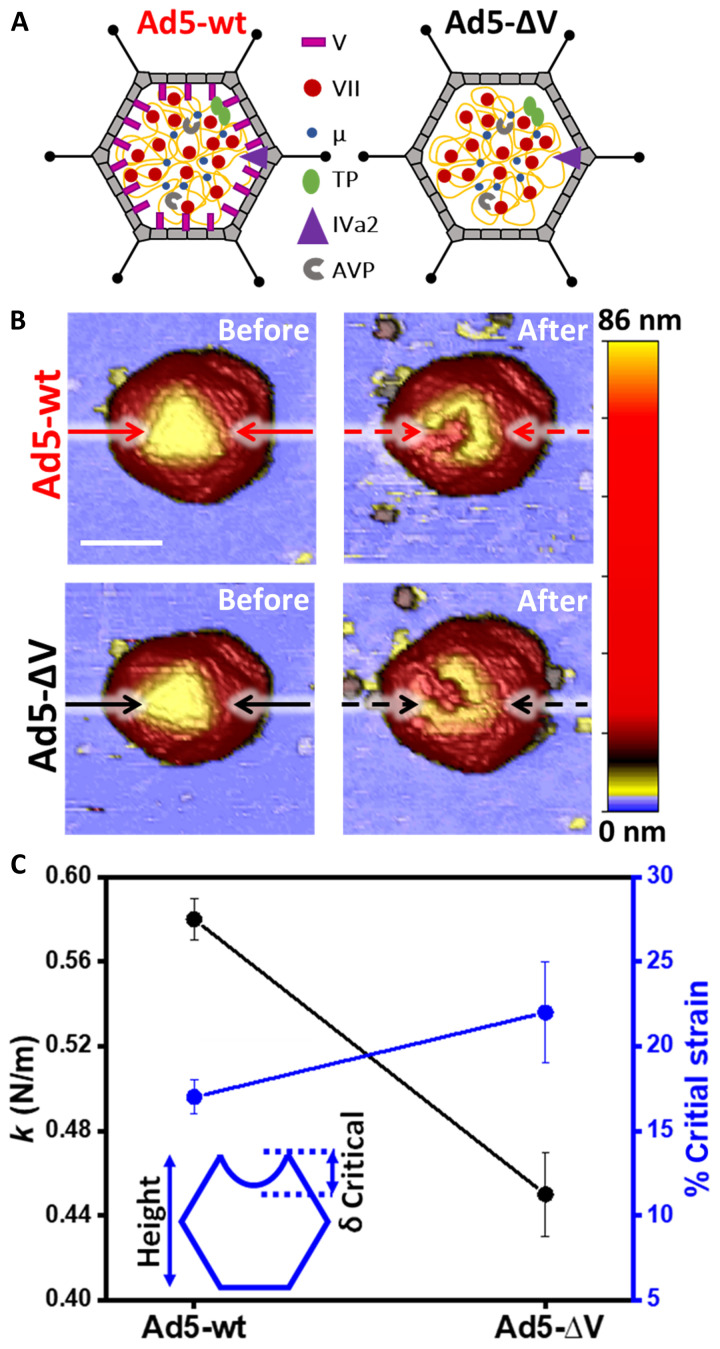
Mechanical properties of Ad5-∆V and Ad5-wt particles. (**A**) Schematic representation of the specimens using different symbols for the proteins present in the core. Adapted from (*2*). (**B**) Representative AFM images of Ad5-wt and Ad5-∆V before (left) and after (right) an indentation experiment. Solid and dashed black and red lines represent profiles traced in Fig. 3A. Scale bar, 70 nm. Images are colored by height, as indicated by the height scale bar at the right-hand side. (**C**) Stiffness (spring constant *k*, left axis, black) and brittleness (critical strain, right axis, blue) of the two specimens. The inset shows a cartoon of critical indentation analysis.

Unlike proteins VII and μ, protein V is not cleaved during maturation and is not conserved throughout the *Adenoviridae* family; it is specific to the Mastadenovirus genus that includes the human adenoviruses (*26*). Cross-linking studies indicated that, in the virion, protein V is in close contact with the other core proteins, VII and μ (*27*). Further, protein V was shown to contact protein VI, located on the inner surface of the icosahedral shell (*27*, *28*). A direct association between V and VI, as well as binding of protein V to dsDNA, has also been demonstrated in vitro using recombinant proteins (*29*). Protein V is released in two steps during uncoating: an early step around endosomal penetration and a terminal uncoating step of the incoming virion at the nuclear pore complex (NPC) (*30*). These observations support the notion that protein V is located at the core periphery linking the Ad5 genome with the capsid (Fig. 1A). The exact location of protein V in the viral particle is not defined, as only a small (25 amino acid) region of the protein has been tentatively assigned to density located on the inner hexon surfaces near the facet center of enteric human adenovirus Ad41 (*31*).

A previous study showed that Ad5 particles lacking protein V (Ad5-∆V) are less thermostable than the wild-type virion (Ad5-wt) and have reduced infectivity due to a defect in genome delivery into the nucleus (*32*). The defect in infectivity is due to the premature release of Ad5-∆V genome in the cytosol before the particle has arrived at the NPC, reflecting particle instability. Moreover, it was shown that ubiquitination of protein V is necessary for protein V release from the capsid and successful translocation of the viral genome across the NPC. Here, we use atomic force microscopy (AFM) in a cell-free system to compare the mechanical stability and rupture of single Ad5-wt, Ad5-ΔV particles, as well as Ad5 particles with a non-ubiquitinatable protein V (Ad5-V-KR) and provide insights into the role of protein V in capsid stabilization, DNA condensation, and genome exit from broken capsids.

## RESULTS

### Absence of protein V softens the Ad5 particle

We first investigated the mechanical properties of Ad5-∆V compared to Ad5-wt (Fig. 1A). Topographical images acquired before particle nanoindentation with the AFM tip revealed intact virus particles (Fig. 1B, left) with a height of ~85 nm, compatible with the Ad5 nominal diameter, for both specimens (*28*). The spring constants measured from the slope of the force-indentation curves (FIC) were *k*_Ad5−wt_ = 0.58 ± 0.01 N/m (mean ± SEM; *N* = 53) for Ad5-wt (*14*) and *k*_Ad5−ΔV_ = 0.45 ± 0.02 N/m (*N* = 30) for Ad5-∆V (Fig. 1C, left axis, black dots), indicating that the absence of protein V softens the adenovirus particle.

We next estimated the critical strain of the particles, defined as the ratio between the critical deformation (the deformation at which the particle breaks, δ_critical_) and the particle height (Fig. 1C, inset), which informs about particle brittleness (*33*). The critical strain was 17 ± 1% for Ad5-wt and 22 ± 2% for Ad5-∆V (Fig. 1C, right axis, blue), showing that the lack of protein V makes the viral particle more deformable before rupture.

### Absence of protein V decreases the mechanical stability of Ad5 particles

**Fig. 2.**
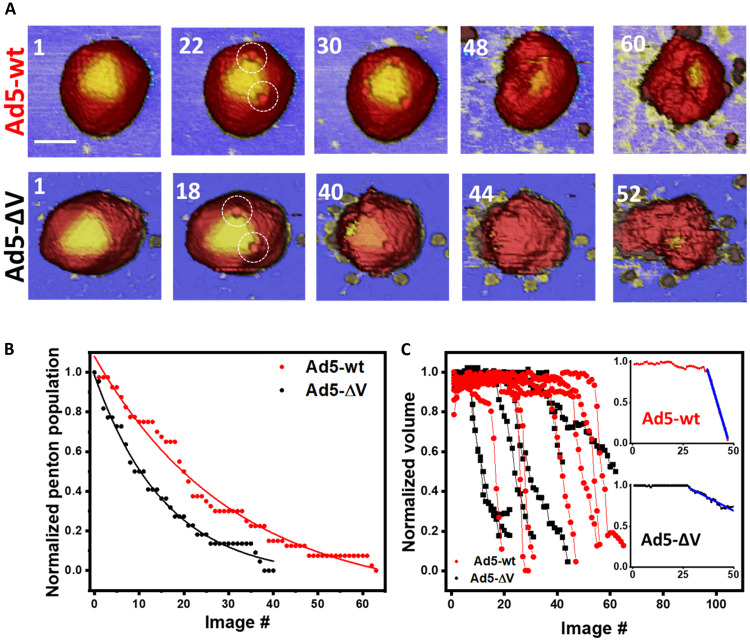
Mechanical stability of Ad5-wt and Ad5-∆V as determined by fatigue assays. (**A**) Examples of Ad5-wt and Ad5-∆V images along a fatigue experiment where the numbers indicate the order of the obtained frames. The imaging time in a single frame is the same for all the experiments and corresponds to ~3 min. Dashed white circles indicate penton vacancies. Scale bar, 60 nm. The color of vertical scale is the same of Fig. 1B. (**B**) Analysis of penton release distribution for seven Ad5-wt and six Ad5-∆V particles (21 pentons and 18 pentons, respectively). The penton release distribution follows an exponential decay. (**C**) Normalized volume evolution along a fatigue experiment for the two specimens studied. The inset shows an example to illustrate the linear fitting (blue line) of the volume loss rate.

Mechanical fatigue assays inform about the mechanical stability of viral particles when subjected to repeated AFM imaging with jumping mode using low force (~100 pN) (*34*). Repeated interaction with the AFM tip triggers adenovirus particle disassembly, starting with release of a single penton, which is composed of penton base and fiber, although fiber cannot be visualized in this setting. Pentons are known to be subjected to extra mechanical stress, thus, in general, they are the weakest elements in an icosahedral capsid and prone to leave the virus particle before hexons (*16*, *20*, *35*–*37*). Other pentons are sequentially lost, followed by capsid cracking and subsequent genome release (*20*, *35*). Figure 2A shows examples of Ad5-wt (top) and Ad5-∆V (bottom) particle uncoating during mechanical fatigue assays. Both particles showed similar topography at the beginning of the experiment, and analogous disassembly patterns as the experiment proceeded (Fig. 2A). First, the visible pentons located at the vertices of the topmost triangular facet were lost (Fig. 2A, white dashed circles), followed by the gradual rupture of the capsid until the particle collapsed. Penton release for all the tested viral particles is shown in fig. S1.

In the example illustrated in Fig. 2A (top), the Ad5-wt particle has lost two pentons after 22 images, and the particle appeared to collapse after 60 images. In the Ad5-∆V example (Fig. 2A, bottom), two pentons popped out after 18 images and particle collapse took place at frame 52. We quantified the mechanical fatigue parameters of seven Ad5-wt and six Ad5-∆V particles. Figure 2B shows the rate of penton loss. We separately normalized the penton population to the total observable pentons for the seven Ad5-wt and six Ad5-ΔV particles studied and plotted the normalized penton population along time. In these assays, time is expressed as number of images (*35*). At the beginning of the experiments, all the pentons were present (normalized population = 1). As pentons were sequentially released, the population decreased until all three pentons located at the visible triangular facet disappeared (normalized population = 0). The penton loss distribution followed an exponential decay, allowing us to estimate the mean lifetime of a penton (*16*), resulting in 26 ± 2 images per penton for Ad5-wt and 16 ± 1 images per penton for Ad5-∆V. This analysis indicates that there is a higher susceptibility to release pentons in the absence of protein V.

We next quantified the volume reduction in viral particles during the fatigue experiments (Fig. 2C). The particle volume in each frame was normalized to its initial value in the first frame of the dataset. For both Ad5-wt (red) and Ad5-∆V (black), particle volume remained almost constant during the first part of the disassembly, where the pentons were lost sequentially. After the three visible pentons were released, the particle volume decreased abruptly in a few frames (see table S1). A linear fitting of the normalized volume data provides the rate of volume loss per frame (Fig. 2C, inset, blue), which was 8 ± 6% frame^−1^ for Ad5-wt and 4 ± 2% frame^−1^ for Ad5-∆V. These values indicate that, contrary to the effect observed for pentons, particles lacking protein V have a lower susceptibility to lose core components than wild-type particles. In line with the latter, also the final normalized volume was larger for Ad5-∆V [0.29 ± 0.19 (mean ± SD)] than for Ad5-wt (0.08 ± 0.04), indicating that the retention of core material is higher in Ad5-∆V.

To evaluate whether the differences in mechanical properties and stability between Ad5-wt and Ad5-∆V could be derived from structural changes induced by the lack of protein V, we obtained a cryo–electron microscopy (cryo-EM) map of Ad5-∆V at 4.4 Å resolution and compared it with an Ad5-wt map of similar quality (Supplementary Methods and fig. S2). No substantial differences were detected that could account for protein V or other changes related with its absence in the icosahedrally ordered capsid. Noticeably, the small density attributed to a central region of protein V in the Ad41 study (*31*) was also present in our Ad5-∆V map (fig. S2C).

### Absence of protein V impairs genome escape from disrupted capsids

**Fig. 3.**
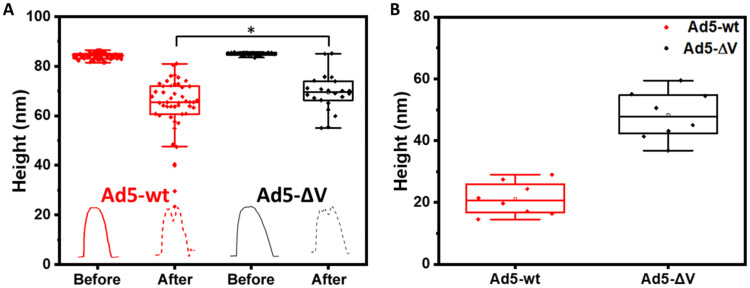
Height analysis after nanoindentation or mechanical fatigue experiments provides information about the core condensation state. (**A**) Height distribution of all the viral particles imaged, before and after a single indentation. The height was measured along a profile passing through the capsid crack, as indicated by the solid and dashed, black and red lines in Fig. 1B. The profiles obtained at Fig. 1B are shown at the bottom of the plot in red and black colors for Ad5-wt and Ad5-ΔV particles, respectively. Before the indentation, the value of height at the profile apex is plotted. After the indentation, we plot the height at the lowest point in the crack. Statistical significance from ANOVA test, *P < 0.05. (**B**) Final height values from all the mechanical fatigue experiments.

AFM images taken before and after a single nanoindentation demonstrated the rupture of the shell, causing a crack and therefore a loss of height (Fig. 1B). By measuring the height at the lowest part of the crack, we obtained an indication of the amount of core contents remaining inside the disrupted capsid (Fig. 3A). Before the nanoindentation, the height value reflects the nominal size for an intact particle (Fig. 3A, before). After the indentation, the crack was deeper in Ad5-wt [red, *h*_Ad5-wt_ = 63.7 ± 1.8 nm (mean ± SEM), *N* = 48] than in Ad5-∆V (black, *h*_Ad5-ΔV_ = 69.4 ± 1.6 nm, *N* = 25). One-way analysis of variance (ANOVA) test at the 0.05 level indicated that population means are significantly different. Therefore, these experiments show that it is more difficult for the AFM tip to penetrate Ad5-∆V than Ad5-wt particles, after the capsid is cracked open. A similar observation was previously used as an indicator of the condensation state of the core (*38*). Alternatively, the difference between crack depth in Ad5-ΔV and Ad5-wt could indicate that the core components escaped more easily from Ad5-wt than Ad5-∆V particles.

To extend our analysis of particle disassembly, we examined the variation in particle height along fatigue experiments (*20*). In Fig. 3B, we plotted the final height for single Ad5-wt (red dots) and Ad5-ΔV (black dots) particles after mechanical fatigue experiments. From these measurements, we estimated the average final height for each type of particle *h*_Ad5-wt_ = 21 ± 5 nm (mean ± SD), *N* = 7 and *h*_Ad5-ΔV_ = 48 ± 7 nm, *N* = 6. This result, together with the slower volume loss in Ad5-ΔV (Fig. 2B), indicates that in the absence of protein V, the core is more condensed and does not easily diffuse out of the capsid, even if the Ad5-ΔV particles lost pentons faster than Ad5-wt.

**Fig. 4.**
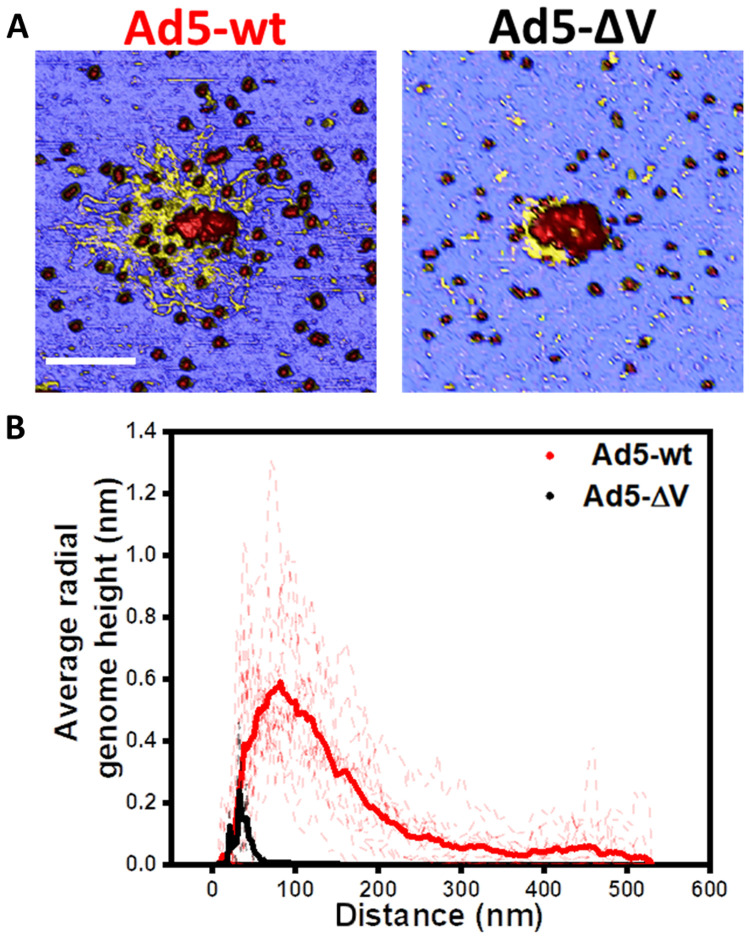
Released genome height after mechanical fatigue assays. (**A**) Examples of AFM image showing Ad5-wt (left) and Ad5-∆V (right) particles disrupted after a mechanical fatigue assay. Scale bar, 200 nm. (**B**) Radial average genome height distribution for Ad5-∆V and Ad5-wt. Solid lines represent the average distribution for each type of specimen.

To further analyze the influence of protein V on adenovirus genome release, we analyzed the debris appearing on the mica surface after the virus particle crumbled down in mechanical fatigue assays (fig. S3). This debris included genome (yellow) (*14*, *39*), capsid pieces, and individual capsomers (dark red blobs) (*14*, *20*). In both Ad5-ΔV and Ad5-wt particles, there were large remnant objects at the center of the image, which corresponds to the particle initial position, surrounded by smaller objects (capsomers) and fibrous material (genome) spreading out from the largest piece (Fig. 4A). For Ad5-wt, released genome was observed in 100% of the experiments, and its diffusion was efficient enough to spread far away on the substrate (Fig. 4A, left). For Ad5-∆V, released capsomers (or other small objects) were also seen to spread away from the particle, but the genome was only seen in 25% of the fatigue experiments, and just a small amount of released fibrous material appeared, very close to the central debris of the capsid (Fig. 4A, right).

We quantified how far the virus genome spread out after escaping the dismantled capsid by calculating a radial average of the topographical height at the end of the fatigue experiment (fig. S4). The radial average depicts the average height of points that are located at the same distance from the center of the virus particle. In Fig. 4B, the radial average of height as a function of distance is shown for each viral particle studied (dashed lines), and the averaged data in solid lines. This chart shows that the genome of Ad5-wt particles reached positions up to 500 nm from the center of the particle, with a peak at 100 nm. However, in Ad5-ΔV the genome hardly spread beyond 60 nm. From these analyses, we conclude that the genome does not escape from Ad5-∆V particles as easily as from Ad5-wt, in line with a higher condensation state.

In a previous study performed on particles lacking the major core protein VII, we devised a method to objectively estimate the thickness of DNA fibers without recoursing to manually defined profiles across the regions of interest. These measurements indicated bundling of the dsDNA molecule in the presence of protein VII (*14*). Since in Ad5-ΔV–disrupted particles the genome does not spread out (Fig. 4 and figs. S3 and S4), it was not possible to carry out the same kind of analysis here. Therefore, we measured genome strand thickness by tracing height profiles in a limited set of images (seven for Ad5-wt and two for Ad5-ΔV) where separated fibers could be observed (fig. S5). By taking the maximum height of several profiles for Ad5-wt and Ad5-ΔV, respectively (fig. S5A, top and bottom), it was possible to obtain a statistical analysis of the thickness of the released fibers for each viral particle. The results of this analysis (fig. S5B) show that the released fibers present a similar average thickness [*h*_Ad5-wt_ = 2.5 ± 0.7 nm (mean ± SD), *N* = 22 and *h*_Ad5-ΔV_ = 2.5 ± 0.4 nm, *N* = 15] in both specimens, close to 2 nm (*40*). One-way ANOVA test indicated that population means were not significantly different. Hence, we did not detect differences in the degree of DNA bundling related to the presence or absence of protein V. In addition, lack of protein V did not noticeably change the DNA packing inside the particle, at least as judged by the lack of concentric shells in icosahedrally averaged cryo-EM maps (fig. S2, A and B).

### Removal of ubiquitin target residues in protein V has an effect on particle stability but not on genome condensation and in vitro release

In a previous study, an Ad5 mutant (Ad5-V-KR) was generated with all 26 lysine residues in protein V changed to arginine, to abrogate ubiquitination during virus entry (*32*). Unlike Ad5-ΔV, Ad5-V-KR particles are stable in the cytoplasm but impaired at viral DNA (vDNA) nuclear delivery. They misdeliver vDNA at the NPC yielding similar ratios of capsid-free cytoplasmic to nuclear genomes as Ad5-∆V (*32*).

**Fig. 5.**
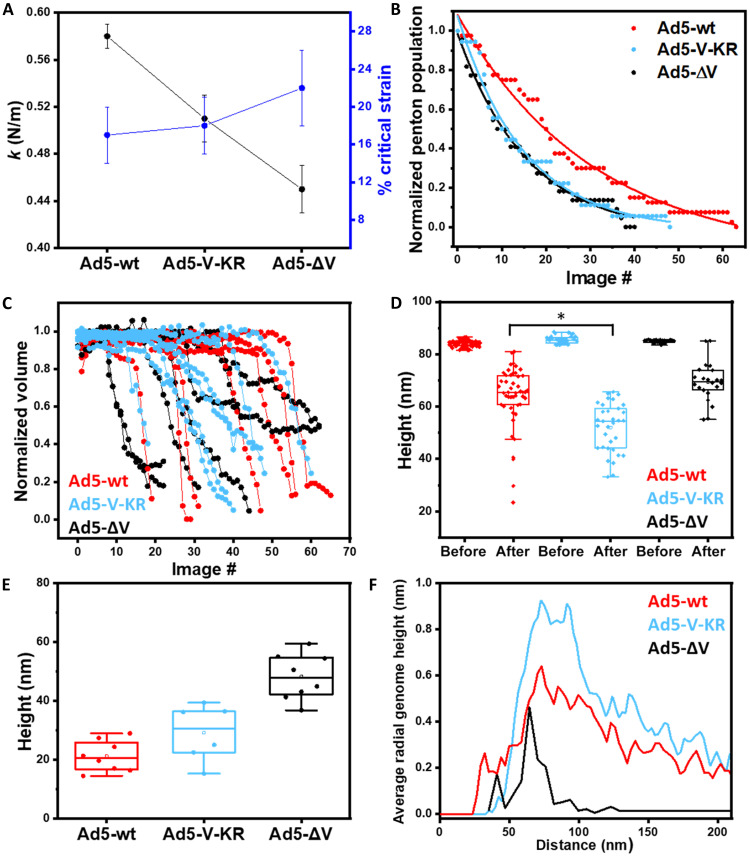
Summary of Ad5-V-KR AFM analyses in comparison with Ad5-wt and Ad5-ΔV. (**A**) Stiffness (spring constant *k*, left axis, black) and brittleness (critical strain, right axis, blue) of the three specimens. (**B**) Analysis of penton release distribution for the three specimens. (**C**) Normalized volume evolution along fatigue experiments for the three specimens. (**D**) Height distribution of all the viral particles imaged, before and after a single indentation. Statistical significance from ANOVA test, *P < 0.05. (**E**) Final height values from all the mechanical fatigue experiments. (**F**) Radial average genome height distribution in 300 nm^2^ images for the three specimens.

At the AFM, an Ad5-V-KR preparation showed a mixed population of intact and pentonless particles and, in some cases, dsDNA laying on the mica surface (fig. S6). Single indentation assays on Ad5-V-KR intact particles (*N* = 36) gave an intermediate stiffness between those of Ad5-wt and Ad5-ΔV (0.52 ± 0.02 N/m) (Fig. 5A). This behavior could reflect a destabilization in the interactions between the capsid and the core when the protein V residues are changed, or it could be related to the impaired integrity of the sample in its initial state, as suggested for other virus preparations independent of protein V mutations (*13*). On the other hand, the Ad5-V-KR brittleness (18 ± 3%) was like that of Ad5-wt, but different to Ad5-ΔV, according to ANOVA test with statistical significance, *P* < 0.05. This result indicates that the modifications in the sequence of protein V do not affect capsid deformability.

In mechanical fatigue experiments, Ad5-V-KR particles (*N* = 6) displayed the typical adenovirus sequential disassembly pattern (fig. S7). Penton loss was fast, with a distribution like that of Ad5-ΔV (mean penton lifetime 16 ± 1 images per penton; Fig. 5B). The volume loss rate was intermediate between Ad5-wt and Ad5-ΔV (volume loss ratio 6 ± 2% frame^−1^; Fig. 5C). In both assays measuring capsid permeability and infectivity, the thermostability of Ad5-V-KR particles was indistinguishable from that of Ad5-wt (fig. S8). Together with the stiffness and critical indentation measurements, these observations indicate that the mere presence of protein V reinforces the capsid, but changes in its sequence impair its mechanical stabilization function.

Measures of the crater height after indentation showed that the AFM tip reached deeper in Ad5-V-KR than in Ad5-ΔV [*h*_Ad5-V-KR_ = 52.1 ± 1.6 nm (mean ± SEM), N = 33] (Fig. 5D). Likewise, the height of particle debris after mechanical disassembly was smaller for Ad5-V-KR than for Ad5-ΔV and close to that of Ad5-wt [*h*_Ad5-V-KR_ = 29.1 ± 9.6 nm (mean ± SD), *N* = 6] (Fig. 5E). We found genome spread on the mica surface in 100% of the Ad5-V-KR mechanical fatigue disassembly assays (fig. S5A, middle), and the genome average thickness [*h*_Ad5-V-KR_ = 2.4 ± 0.5 nm (mean ± SD), *N* = 20] was similar in the three specimens studied (fig. S5b). Last, radial average genome height distributions at the end of disassembly were similar for Ad5-wt and Ad5-V-KR (Fig. 5F). All these results indicate that the Lys to Arg substitutions in protein V had no effect on genome condensation or diffusion out of the capsid.

## DISCUSSION

AFM and nanoindentation studies have found that mechanical properties of viruses depend on various factors such as the amount of genome packaging in bacteriophagesϕ29 or Lambda (*41*, *42*), the DNA-capsid interaction in the minute virus of mice (*43*), the maturation state in bacteriophage HK97 and P22 (*44*, *45*), or the ionic environment in hepatitis B virus (*46*). In the case of adenovirus, core proteins play an important role in virion stability and uncoating, as shown in previous studies on immature particles (*12*, *13*, *20*, *21*, *23*) and particles lacking the major core protein VII (*14*, *16*). Particles lacking protein VII are stiffer and less thermostable than Ad5-wt due to lower genome condensation and the higher internal pressure caused by DNA-DNA repulsion in the absence of the major core protein DNA-condensing activity (*14*, *16*). Immature adenovirus particles, such as the Ad2 *ts1* particles lacking proteolytic processing (*47*), are softer and more thermostable than the mature ones, due to a decrease in internal pressure presumably caused by the enhanced DNA condensing ability of the uncleaved proteins VII and μ (*13*, *21*). This is supported by the observation that the final debris height after mechanical disruption is larger for immature particles than for mature virions, indicative of a condensed core attached to capsid fragments after cracking open of the protein shell, and by cryo-EM images showing the core as a sphere attached to capsid fragments in disrupted Ad2 *ts1* particles (*12*, *20*, *21*).

Protein V binds DNA and has a considerable positive charge (*2*), suggesting that it could help to condense the Ad5 genome. Therefore, we anticipated a higher internal pressure and a higher stiffness in the Ad5-ΔV particles, as was previously found for particles lacking core protein VII (*14*). However, we observed rather the opposite: particles lacking protein V were softer and less brittle than Ad5-wt. Moreover, in the absence of protein V, the spring constant (*k*_Ad5−ΔV_ = 0.45 ± 0.02 N/m) was similar to that of empty Ad5 particles (*k*_E_ = 0.44 ± 0.03 N/m), and lower than that of the immature particles produced by the Ad2 *ts1* mutant, which contain the full genome and all core proteins, among them VII and μ in their precursor version (*k*_ts1_ = 0.49 ± 0.04 N/m) (*13*, *14*, *22*, *25*, *47*). This result indicates that rather than decreasing the internal pressure in the virion, protein V reinforces the adenovirus capsid. The reinforcement can be related to the larger volume occupied by the genome and proteins inside Ad5-wt, or to an architectural effect related to the position of protein V on (or near) the inner capsid surface. The capsid reinforcement effect extends to penton stabilization, as shown by the mechanical fatigue assays where Ad5-ΔV particles lost pentons faster than Ad5-wt (Fig. 2B). This early loss of pentameric capsomers is in agreement with the lower thermostability of Ad5-ΔV as determined by the binding of a capsid impermeable fluorescent dye to the viral genome (*32*). The mechanical stability and stiffness differences between Ad5-wt and Ad5-ΔV do not correlate with architectural differences in the organization of the icosahedral shell, as shown by our cryo-EM analysis (fig. S2). Further, the density tentatively assigned to protein V in Ad41 (*31*) is also present in our Ad5-ΔV map, suggesting that either this assignment is incorrect, or some other capsid component fills the same binding pocket when protein V is absent.

**Fig. 6.**
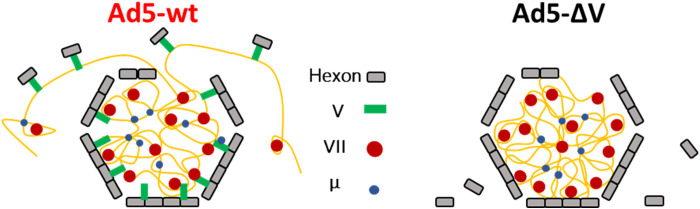
Model for the role of protein V in facilitating Ad5 genome release. In Ad5-wt (left), all the core proteins are present. In this situation, proteins VII and μ condense the genome, while protein V makes contacts with the core and the capsid, reducing the DNA condensation and dragging the genome out when capsomers are released. In Ad5-∆V (right), the condensing action of protein VII and μ is not antagonized by protein V pulling the DNA toward the capsid, so that the genome is more strongly condensed and remains inside the disrupted particle.

Although Ad5-ΔV particles readily lose pentons, the Ad5-ΔV cracked particles were less prone to release their contents than Ad5-wt, indicating a more condensed core (Figs. 2C to 4). While this observation contradicts the expected DNA condensation role of protein V, it is in line with the unchanged thickness of the few DNA fibers expelled on the mica surface compared to Ad5-wt particles (fig. S5). This similarity indicates that lack of protein V did not affect the short-range condensation exerted by the other core proteins (VII and μ). We interpret these observations in the light of the capsid-core bridging function of protein V. The capsid-genome connections mediated by protein V would pull the DNA toward the inner capsid surface and thereby exert a decondensing action opposite to that of proteins VII and μ. When the Ad5-wt particle breaks, protein V bound to the released capsomers would drag the genome outward, thereby enhancing the DNA separation from the disrupted capsid. In Ad5-ΔV particles, however, capsomers are free to leave without forcing the condensed DNA-protein core to be released (Fig. 6).

In this single particle study, we find that in the absence of protein V, the genome does not readily diffuse away from the disrupted capsid. At first sight, this result seems to disagree with the situation in cells, where the incoming virion DNA readily separates from the Ad5-ΔV particles in the cytosol before reaching the NPC (*32*). Premature genome loss in cells is not observed with Ad5-wt, indicating that, in cells, protein V has an important function in securing the stability of the incoming Ad5 particles up to the point when the particles attach to the NPC and are triggered to disassemble by the catalytic activities of the E3 ubiquitin ligase Mib1 and the kinesin motor protein (*49*–*52*).

The single virion situation and the in cellula situation are different in many ways. For example, both kinesin and dynein motor proteins attach to the incoming Ad5 particle and engage the virions in bidirectional traffic on microtubule tracks (*52*–*56*). It is possible that the Ad5-ΔV particles become unstable in the cytosol due to molecular crowding and that capsid fragments separate from the virion DNA by cytoplasmic transport processes absent in the AFM experiments. In the AFM setup, the particle is in a highly diluted environment of an ionic protein-less buffer and statically adsorbed to the mica surface, a situation which may limit the separation of the DNA core from the broken capsid.

However, the AFM experiments clearly showed that the core is more compact in the absence than in the presence of protein V. This situation could be physiologically relevant, as the incoming Ad5 particle when docked at the NPC receives a trigger from Mib1 to ubiquitinate and degrade protein V before the vDNA is imported into the nucleus (*30*, *32*, *50*, *57*). The present work underlines the earlier notion that the DNA core lacking protein V is a better substrate for nuclear import than the DNA core containing nondegradable protein V (*32*). Replacing lysine residues with arginine in protein V does not seem to be critical for genome condensation, as shown by our results on the Ad5-V-KR mutant particle. However, it may have some effect on mechanical reinforcement, causing a stiffness decrease and penton instability. These observations corroborate the notion that the defect in Ad5-V-KR infectivity is due to the lack of ubiquitination of protein V [as shown in (*32*)], which precludes its removal from the particle at the NPC. Removal of protein V would be required for core condensation at the NPC facilitating genome import into the nucleus.

In summary, we have shown here that protein V influences the mechanical stability and disruption of human adenovirus particles. In the absence of protein V, adenovirus particles become softer and less brittle but are more prone to lose pentons. These results indicate a mechanical reinforcement role for protein V, consistent with its location on the inner capsid surface. Moreover, mechanical fatigue assays showed that in the absence of protein V, the core contents are kept inside cracked capsids, and the genome does not diffuse as easily as in the Ad5-wt particles. Together, these results suggest that protein V is not condensing the DNA. On the contrary, by establishing bridges between core and capsid, protein V exerts a genome decondensing action antagonistic to that of the other core proteins VII and μ. This effect presumably facilitates the release of the Ad5 genome when the capsid is fragmented at the NPC and protein V is detached from the virion. We speculate that the degradation of protein V from broken particles by Mib1-mediated ubiquitination and proteasomal degradation separates the vDNA from capsid fragments and thereby provides a genome structure favorable for nuclear import. Notably, the incoming hexon is not found to be imported into the nucleus, and its binding partner protein VI, which mediates hexon import during virus assembly (*58*), is completely degraded during entry (*59*–*61*).

In conclusion, this study underscores the importance of elucidating physical properties of single virions to better understand the mechanisms of virus entry. It extends an increasing number of studies linking physical properties of virions to cell entry and infection processes and provides tangible hypotheses for virology, vectorology, and synthetic biology (*13*, *14*, *16*, *20*, *21*, *35*, *62*–*69*).

## MATERIALS AND METHODS

### Virus preparation

#### Ad5-wt

The E1-deleted Ad5/attP vector was used as wild-type Ad5 in this study (*70*). Ad5/attP was propagated in human embryonic kidney–293 cells at 37°C and harvested 36 hours after infection. Subsequently, cells were pelleted by centrifugation, medium was removed, and cell pellets were flash-frozen in liquid nitrogen for storage. Particles were then purified by equilibrium centrifugation in two consecutive CsCl gradients, desalted on a Bio-Rad 10DG column, and stored in Hepes Buffered Saline (HBS), [20 mM Hepes (pH 7.8) and 150 mM NaCl] supplemented with 10% glycerol for long-term storage at −80°C. The data for Ad5-wt have been reported previously (*14*, *16*).

#### *Ad5*-Δ*V and Ad5-V-KR*

These viruses were designed and grown as previously described (*32*). Briefly, protein V was deleted from the genome by recombineering from the pKSB2 bacmid, which contains the entire Ad5-wt300 genome (*71*, *72*). The resulting virus was expanded in 911 cells at 37°C and harvested 5 days after infection. The supernatant and cellular fractions were separated through centrifugation. Particles within the supernatant were polyethylene glycol (PEG) precipitated, in 0.5 M NaCl and 8% (w/v) PEG8000, overnight at 4°C. Pelleted cells were lysed by three freeze-thaw cycles and lysates stored at −80°C. Particles were purified over two CsCl gradients (*25*, *60*), extracted and dialyzed on a Slide-A-Lyzer Dialysis Cassette (Thermo Fisher Scientific) against dialysis buffer [150 mM NaCl, 1 mM MgCl_2_, and 10 mM tris/HCl (pH 8.1)] supplemented with 10% glycerol for long-term storage at −80°C.

### AFM experiments

All measurements were conducted with an AFM (Nanotec Electrónica S.L., Madrid, Spain) operated in Jumping Mode (*34*). Rectangular silicon nitride cantilevers (RC800PSA, Olympus, Tokyo, Japan) with nominal spring constants of 0.05 N/m were used and routinely calibrated using the Sader’s method (*73*). The topographical images were acquired, and the data were analyzed using the WSxM software (*74*).

Virus samples were dialyzed against HBS for 1 hour at 4°C to remove glycerol and equalize buffer conditions. Sample aliquots (5 μl) were diluted to 20 μl in HBS supplemented with 5 mM NiCl_2_, to obtain a final concentration of 2 × 10^11^ viral particles (vp)/ml for Ad5-wt and 2.25 × 10^11^ vp/ml for Ad5-ΔV. Samples were incubated for 20 min on freshly cleaved muscovite mica and washed five times with the same buffer to remove the nonadsorbed particles. After washing, a final drop of 500 μl HBS + 5 mM NiCl_2_ was added to the sample, and a drop of 20 μl was added to the tip.

For single nanoindentation assays, individual particles were deformed with the AFM tip, and FIC were recorded. Virus topography was monitored before and after recording each FIC. The particle mechanical properties [elastic constant (*k*) and critical strain (δ_critical_)] were obtained from these FIC. For mechanical fatigue assays, topographical images were consecutively acquired on the same virus particle with 128 × 128 pixels spanning an area of 300 nm^2^, at a constant force (100 pN). With this approach, the disassembly process of each viral particle can be tracked to obtain the virus volume and height (*20*). Mechanical fatigue conditions, including force and imaging time, are kept constant in all the experiments for the sake of comparison. The volume of each particle was calculated using the flooding tool in WsXM. Flooding sets all the image values below a given threshold to a constant value, allowing the calculation of parameters such as the number, surface, perimeter, and volume of the particles represented by the nonflooded pixels. To consider the viral particle debris, we set a minimum height threshold of 15 nm.

## Notes

View/request a protocol for this paper from *Bio-protocol*.
