# Supplementary material for: Adenovirus core protein V reinforces the capsid and enhances genome release from disrupted particles
Source: Sci Adv. 2023 Apr 7;9(14):eade9910. doi: 10.1126/sciadv.ade9910 (PMC10081844; doi:10.1126/sciadv.ade9910)
Supplement: Supplementary file 1 — Supplementary Methods Figs. S1 to S8 Table S1 [file sciadv.ade9910_sm.pdf]

Science Advances
AAAS

Supplementary Materials for

Adenovirus core protein V reinforces the capsid and enhances genome release from disrupted particles

Natalia Martín-González *et al.*

Corresponding author: Pedro J. de Pablo, p.j.depablo@uam.es

Sci. Adv. **9**, eade9910 (2023)
DOI: 10.1126/sciadv.ade9910

This PDF file includes:

Supplementary Methods
Figs. S1 to S8
Table S1

Supplementary Methods

Cryo-electron microscopy

Ad5-ΔV purified virus particles ($9x10^{11}$ particles/ml) were dialyzed for 1 h at 4 °C against phosphate buffered saline (PBS, 137 mM NaCl, 2.7 mM KCl, 10 mM Na_2HPO_4, 1.8 mM KH_2PO_4 at pH 7.4) to remove the storage glycerol and vitrified in glow discharged Quantifoil R2/2 300 mesh Cu/Rh grids using a Leica CPC plunger. Particle concentration was increased by consecutively incubating the grid on 5 drops of the sample before the final blotting and plunging in liquid ethane (*77*). Cryo-EM images (2106 movies) were recorded in a 200 kV Talos Arctica microscope equipped with a Falcon II detector, with a total dose of 50 e-/$Å^2$ distributed over 50 frames, at a nominal pixel size of 1.42 Å and a defocus range between -0.5 and -3 µm.

All image processing and 3D reconstruction tasks were performed within the Scipion framework (*78*). Frames were aligned using Motioncor2 and weighted according to the electron dose received before averaging (*79*). The CTF was estimated using CTFFIND4 (*80*). Particles (8151) were semi-automatically picked from micrographs, extracted into 950×950 pixel boxes, normalized and resized to a sampling rate of 2.08 Å/px (640 px box size), using Xmipp(*81*). All 2D and 3D classifications and refinements were performed using RELION(*82*), as previously described (*14*). Icosahedral symmetry was imposed throughout the refinement process. The initial reference for 3D classification was a lizard adenovirus cryo-EM map (*83*), low-pass filtered to 60 Å resolution. The class yielding the best resolution, containing 6550 particles, was individually refined using the map obtained during the 3D classification as a reference. Anisotropic magnification and per particle defocus corrections, followed by a new 3D refinement and Ewald sphere correction, were carried out using RELION version 3.1.2 (*84*). Final map resolution was 4.4 Å, as estimated according to the gold-standard FSC = 0.143 criterion implemented in RELION auto-refine and postprocess routines (*85*). The actual map sampling was estimated by comparison with a human adenovirus high resolution model (PDB ID 6B1T) (*28*) in UCSF ChimeraX (*86*), yielding a value of 2.025 Å/px. For comparison, a similar size group of particles (6481 particles) was randomly extracted from a previous Ad5-wt

dataset collected under similar conditions (*14*, *16*), and processed as described above, yielding a final map at 5.0 Å resolution. No map sharpening procedures were used. The Ad5-V cryo-EM map is deposited at the Electron Microscopy Data Bank (EMDB; www.ebi.ac.uk/pdbe/emdb) with accession number EMD-15694.

Xmipp was used to calculate radial average profiles, which were rescaled to a common value range in the [-1, 1] interval within a region comprising the hexon shell (radii 315 to 464 Å). Remnant maps for both Ad5-wt and Ad5-ΔV were calculated by masking off the density closest to the Ad5-wt molecular model (*28*) using ChimeraX.

Thermostability assays

Thermostability assays were conducted as previously described (*32*). One microgram of purified virus particles was incubated in a thermocycler for 5 min at 40°, 43°, 47°, 50°, 53°, 57°, or 60°C. Samples were cooled down on ice and incubated with 5 µM DiYO-1 (AAT Bioquest) for 5 min at room temperature. Fluorescence was recorded with a Tecan Infinite M200 plate reader (excitation, 490 nm; emission, 520 nm).

To assess the influence of heat treatment on infection, 10,000 A549 cells were seeded in a black 96-well imaging plate. On the following day, virus suspensions were heat-treated for 5 min at 40°, 40.3°, 40.9°, 42°, 43.4°, 44.8°, 46.2°, 47.6°, 49°, 50°, or 80°C. Samples were placed in DMEM (supplemented with 2% FCS, non-essential amino acids, and penicillin/streptomycin) and added to the cells. After 24 hours, cells were fixed, quenched, and permeabilized. Cells were stained with rabbit anti–protein VI (*59*) and goat anti-rabbit Alexa Fluor 488 (Thermo Fisher Scientific, #A-11008) in blocking buffer (10% goat serum in PBS) containing DAPI. Cells were imaged in an IXMc high-throughput microscope in wide-field mode with a 20x objective. Nuclei were segmented using CellProfiler according to the DAPI signal, and infection was quantified on the basis of the protein VI signal over segmented nuclei.

Supplementary Figures

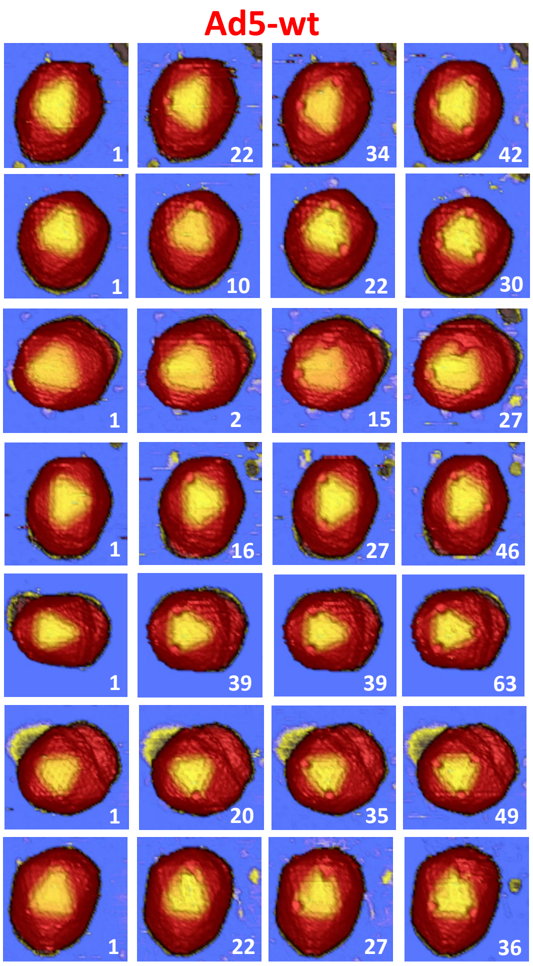

Figure S1. Penton release in mechanical fatigue experiments. Topografical frames in where the pentons are released for seven Ad5-wt (left) and six Ad5-ΔV (right). Frame number is shown in each image. Scale bar corresponds to 45 nm.

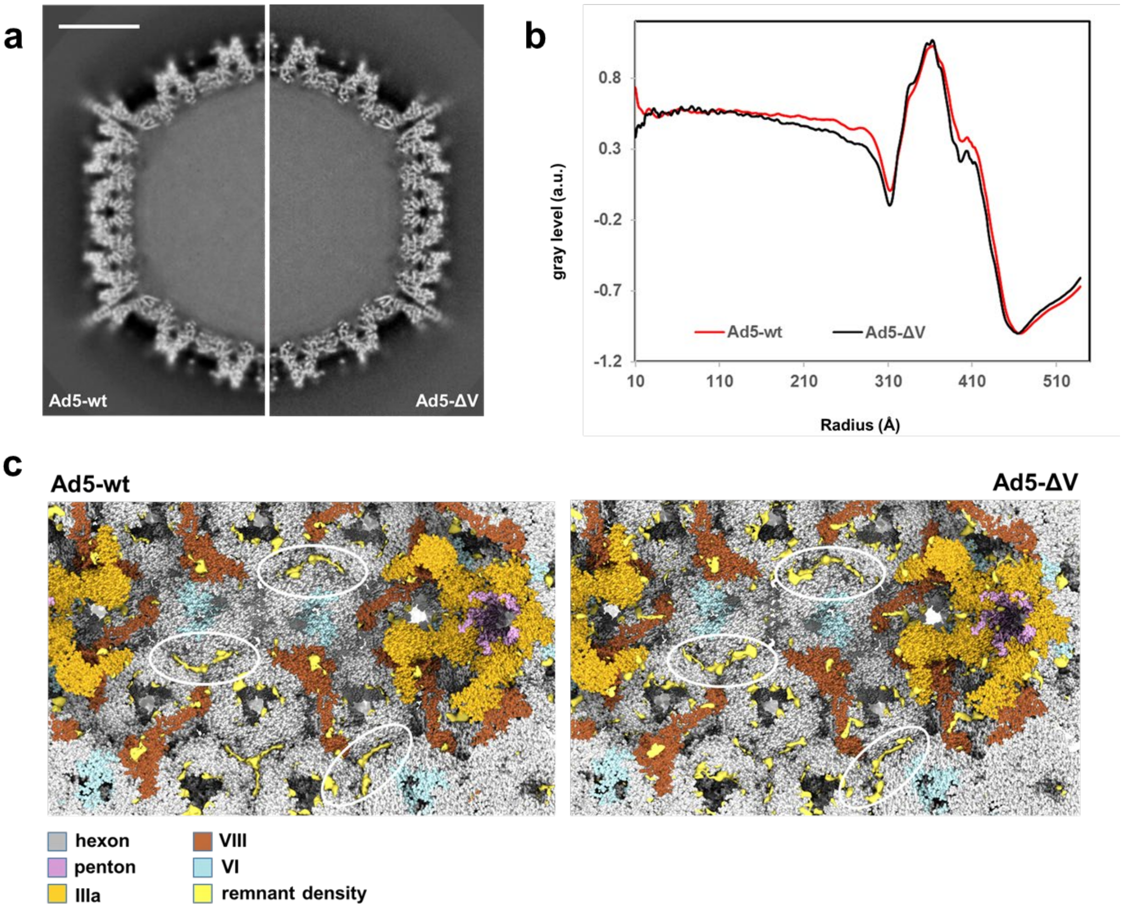

Figure S2. Comparison of Ad5-wt and Ad5-ΔV cryo-EM maps. (a) Central sections of the cryo-EM maps. The bar represents 20 nm. Higher density is shown in white. (b) Radial average profiles of the Ad5-wt and Ad5-ΔV maps. (c) Comparison of the Ad5-wt and Ad5-ΔV remnant maps. Molecules traced in (*28*) are shown in different colors, according to the legend. Density where no models were traced (remnant density) is in yellow. White ovals indicate densities attributed to protein V in (*31*). The view is along a 2-fold symmetry axis from inside the particle.

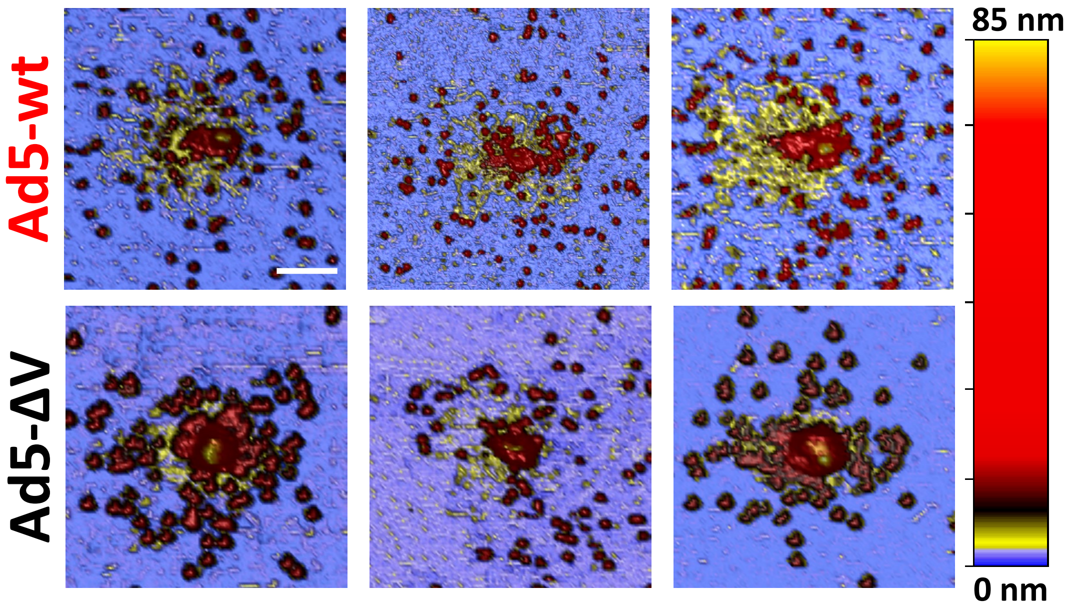

Figure S3. Final topography of adenovirus particles after mechanical fatigue experiments. Three topography examples images of 750 nm^2 showing the genome released (yellow fibers) after mechanical fatigue experiments in Ad5-wt particles (top) and Ad5-ΔV (bottom). Scale bar corresponds to 150 nm. The color bar at the right side represents the height distribution of the topographical images.

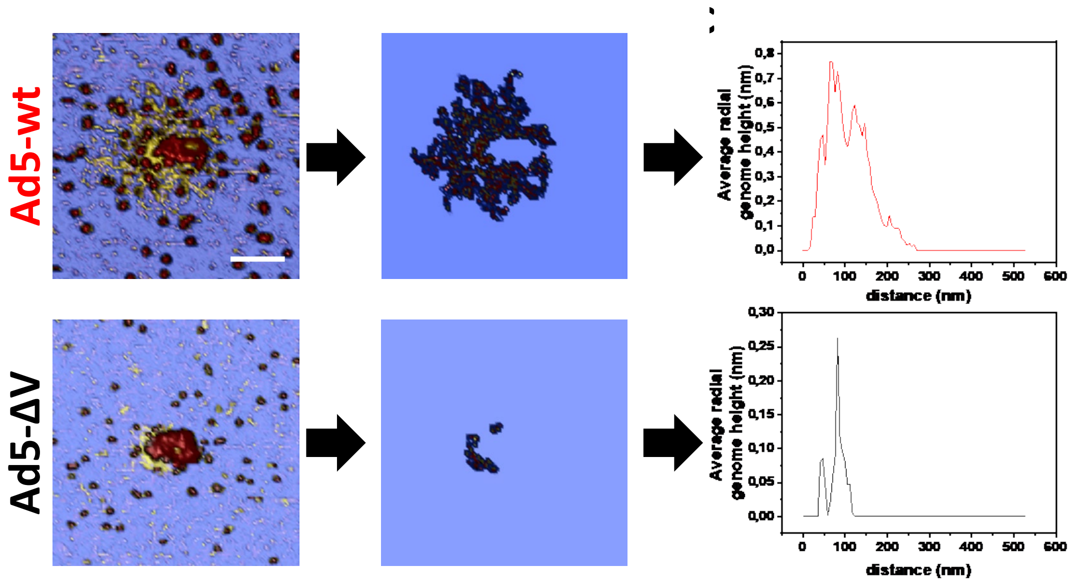

Figure S4. Average radial genome-height analysis. (a) Images showing the complete recorded field of view after a mechanical fatigue experiment. The scale bar corresponds to 150 nm. (b) Flooded image to isolate the genome contribution (height 2 to 4 nm). (c) Radial average of the genome height for the example particles shown in the images (Ad5-wt in red, Ad5-ΔV in black) The radial average consists of estimating the average height of points that are located at the same distance from the center of the virus particle. The center is defined by the maximum height points of the particle debris. The analysis was performed with WsXM software (http://www.wsxm.eu/). The average result for all particles studied is shown in Figure 4b.

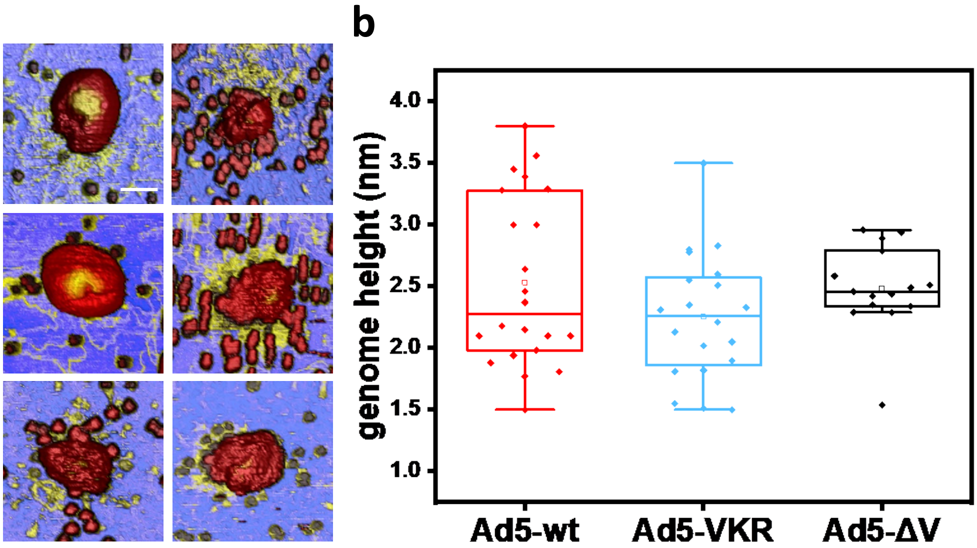

Figure S5. Analysis of released genome fibers upon mechanical fatigue of virions. (a) Example images showing genome release along a mechanical fatigue assay. The scale bar corresponds to 60 nm. (b) Genome height distribution measured at profiles traced across the yellow strands (released filaments).

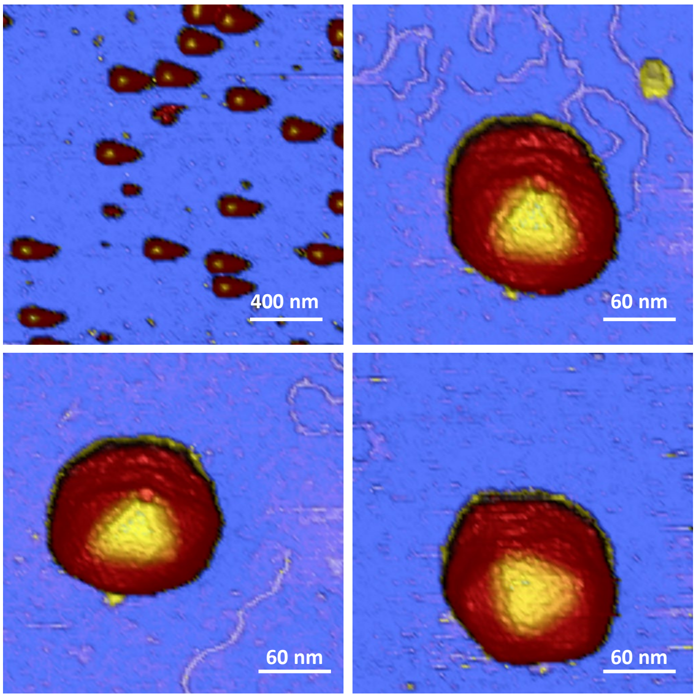

Figure S6. Example AFM images of Ad5-V-KR particles. Top left: general field of view containing several particles. Top right and bottom left: Particles with missing pentons and DNA strands on the mica surface. Bottom right: intact particle.

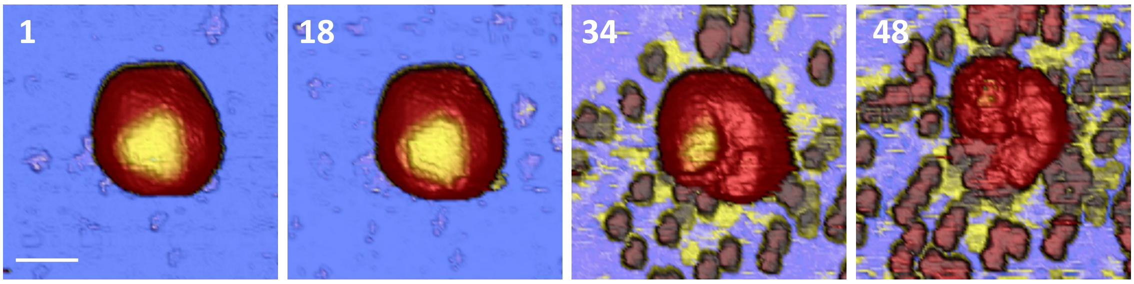

Figure S7. Mechanical fatigue assay of an Ad5-V-KR particle. Images taken along a fatigue experiment are shown, where the numbers indicate the order of the obtained frames. In frame #18 the particle has lost pentons; in frame #34 genome (yellow) is observed on the mica surface, and in frame #48 the particle has collapsed. The scale bar corresponds to 60 nm.

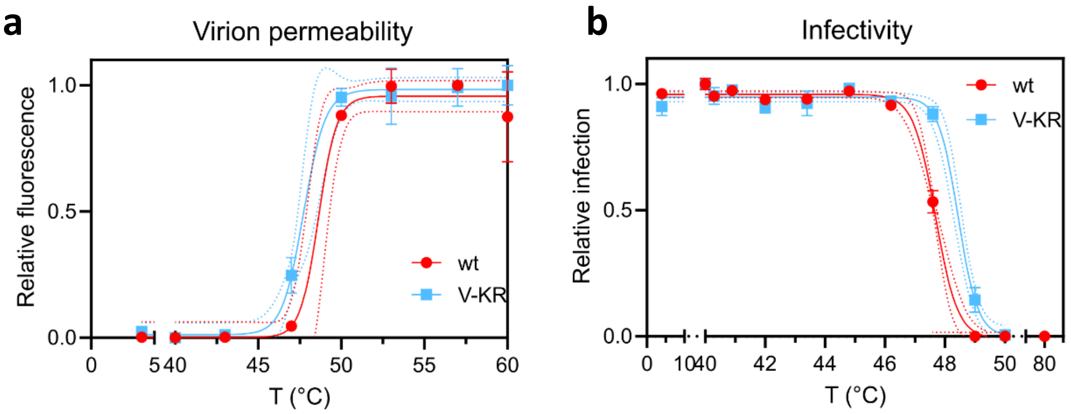

Figure S8. Thermostability of Ad5-V-KR particles *in vitro* and *in cello*. (a) Normalized fluorescence of DiYO-1 after incubation with heat-treated Ad5-wt and Ad5-V-KR particles. Virions were kept at 4°C or subjected to indicated temperatures for 5 min, chilled on ice, incubated with DiYO-1 (5 µM, 5 min), and analyzed by fluorimetry. Means ± SD represent data normalized to the maximum fluorescence (60°C). (b) Normalized infection index in A549 cells of heat-treated Ad5-wt and Ad5-V-KR particles. A549 cells were incubated with heat-treated virus for 24 h, fixed, immunostained for protein VI, labeled with DAPI, and scored for VI-positive nuclei relative to non-treated virus. Means ± SD represent data normalized to the maximum infection index obtained with virus kept at 4°C.

Supplementary Table

Type of viral particle	Particle number	Frame at which three pentons are lost	Initial volume (nm³)	Volume in frame at which three-pentons are lost (nm³)	Final volume (nm³)	% of volume variation (initial/pentons lost)	% of volume variation (pentons lost/final)
**Ad5-wt**	1	42	780037	723335	36645	-7,27	-94,93
	2	27	1013960	899925	75572	-11,25	-91,60
	3	46	778590	724034	104767	-7,01	-85,53
	4	63	732475	726609	99864	-0,80	-86,26
	5	29	901245	751926	72816	-16,57	-90,32
	6	49	732624	711787	102576	-2,84	-85,59
	7	36	805867	778853	442339	-3,35	-43,21
**Ad5-ΔV**	1	38	654933	544850	196599	-16,81	-63,92
	2	20	786590	736090	36909	-6,42	-94,99
	3	5	975055	939085	301263	-3,69	-67,92
	4	17	889272	834758	152159	-6,13	-81,77
	5	7	1047040	876977	187942	-16,24	-78,57
	6	41	1378380	1134400	689096	-17,70	-39,25

Table S1. Changes in particle volume during a mechanical fatigue experiment. The third column shows the first frame at which loss of the three visible pentons was observed. The rest of the columns indicate the initial volume, volume measured at the frame with three penton vacancies, final volume, percentage of volume variation until the three pentons are released, and percentage of volume variation from this moment till the end of the experiment.
